# Two genomes of the white perch (*Morone americana*), an ecologically important teleost

**DOI:** 10.1093/jhered/esaf034

**Published:** 2025-05-30

**Authors:** Josephine R Paris, Megan A Criss, Jessica L Walsh, Joan Ferrer Obiol, Christopher S Murray, Jason Q Boone, Ann M Petersen

**Affiliations:** Industrial Economics, Incorporated (IEc), Cambridge, MA, United States; Floragenex, Inc., Beaverton, OR, United States; Floragenex, Inc., Beaverton, OR, United States; Department of Biology, Colorado State University, Fort Collins, CO, United States; Biology Department, Woods Hole Oceanographic Institution, Woods Hole, MA, United States; Floragenex, Inc., Beaverton, OR, United States; Industrial Economics, Incorporated (IEc), Cambridge, MA, United States; National Oceanographic and Atmospheric Administration, National Marine Fisheries, Northeast Fisheries Science Center, JJ Howard Marine Laboratory, Sandy Hook, New Jersey, United States; Gloucester Marine Genomics Institute, Gloucester, MA, United States

**Keywords:** aquatic, bioindicator, hybridization, invasive, Moronidae, reference genome

## Abstract

The white perch, *Morone americana*, is a widely distributed and ecologically important teleost native to the east coast of North America. Due to its ease of capture and high abundance across a range of ecological conditions, the white perch has been used – and continues to be developed – as a bioindicator of contaminant exposure. Outside of its native range, the white perch is invasive, negatively impacting local ecology and hybridizing with congeneric species. Using PacBio HiFi data, we present two (female and male) highly contiguous genome assemblies. The female assembly spans 694 Mb (264 contigs, N50: 24.9 Mb), with 85% of the total assembly size captured in the largest 24 contigs (the karyotype for the species is 2*n* = 48). The male assembly spans 688 Mb (265 contigs, N50: 26.4 Mb), with 89% of the total assembly size captured in the largest 24 contigs. Both assemblies have high BUSCO completeness scores of 98.7% (female) and 98.8% (male), and a high *k*-mer completeness (>98% for both genomes). Combining evidence derived from transcriptomic data and a large protein database, we constructed a high-quality annotation for the female assembly (99% BUSCO completeness, 87% OMArk completeness), including 20,699 predicted protein-coding genes, of which 20,406 have a functional annotation and 16,187 have an associated gene name. These reference genomes will support the development of the white perch as a bioindicator and will serve as an important resource for studying the species’ invasiveness and monitoring intraspecific hybridization using genomic tools.

## Introduction

**Fig. 1.**
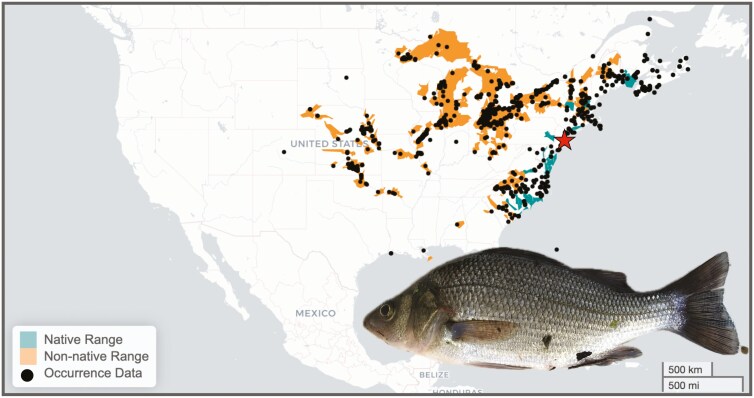
Distribution and typical appearance of the white perch, *Morone americana*. Map of North America showing the native range (in blue) and non-native range (i.e., invasive, in orange). The native range includes the Atlantic Slope drainages from the St. Lawrence-Lake Ontario drainage, Quebec, south to the Pee Dee River, South Carolina (Page & Burr, 1991). Populations in the Lake Ontario drainage probably became established following the construction of the Erie Canal (Fuller et al., 2023). The red star indicates the geographic location (Mullica River, New Jersey) of the samples collected for the generation of the genomes. Range data were provided by the U.S. Geological Survey, 2024, Specimen observation and range data for *Morone americana* (Gmelin, 1789), Nonindigenous Aquatic Species Database, Gainesville, FL, nas.er.usgs.gov/viewer/omap.aspx?SpeciesID = 777, Access Date: 12/18/2024. The image of *Morone americana* is a typical adult collected from the Mullica River, NJ, USA. Photo credit: Ann Petersen.

The white perch, *Morone americana*, is a widely distributed and ecologically important teleost native to the Atlantic coastal region of the United States and Canada (Fuller et al., 2023; Fig. 1). The white perch is not a true perch, but is part of the temperate bass family, Moronidae. It is a semi-anadromous omnivorous species, primarily found in rivers, estuaries, and bays (Kerr & Secor, 2012), where it feeds on benthic invertebrates and smaller fishes (St-Hilaire et al., 2002; Weis, 2005). In turn, white perch are prey for some fish and bird species (Willard, 1977; Margulies, 1990; Walter & Austin, 2003; Hoyle et al., 2017; Rattner et al., 2018). The white perch is important for both recreational and commercial fishing (Fuller et al., 2025).

Due to the white perch’s high abundance, broad distribution, and ease of capture, it has been used as a valuable model to assess environmental contaminant exposure (e.g. NOAA 2024) through both laboratory toxicity testing (Morgan et al., 1973; Richardson et al., 1983; Monosson et al., 1994) and field monitoring programs (Kavanagh et al., 2004; Weis & Ashley, 2007; Matsche et al., 2023). The white perch serves as a bioindicator species in Mid-Atlantic coastal estuaries, particularly in the New York and New Jersey Harbor regions, where there is ongoing remediation of legacy contamination (King et al., 2004; Olson & Tharp, 2020; Smith, 2021). Developing genomic resources for ecologically important species is essential for understanding how contaminants influence eco-evolutionary outcomes and subsequently, how this affects food-web dynamics and ecosystem stability (Whitehead, 2014; Rivkin et al., 2019).

Outside of their native range, white perch are invasive in the imperiled Great Lakes and Mississippi watersheds (Boileau, 1985; Fuller et al., 2025; Irons et al., 2002; Schaeffer & Margraf, 1987; Fig. 1). In these regions, they compete with native species for resources (Feiner et al., 2013), disrupt food web integrity (Munawar et al., 2005), and prey on the eggs of native fish species, such as the walleye (*Sander vitreus*; Schaeffer & Margraf, 1987). Where they are invasive, white perch are also known to hybridize with congeneric species, such as the white bass (*Morone chrysops*) and yellow bass (*Morone mississippiensis*; Todd, 1986; Waldman & Bailey, 1992; Irons et al., 2002). Monitoring of hybrid populations has typically relied on phenotypic differences (Kerby, 1979; Harrell & Dean, 1988) and DNA fingerprinting (Tseng et al., 2000). However, white perch are visually similar to white bass (Fuller et al., 2023), and identifying hybrids using low-resolution markers can be unreliable (Twyford & Ennos, 2012). The development of a reference genome for the species will permit the development of high-sensitivity genomic markers to monitor hybridization dynamics and to evaluate the evolutionary and conservation implications of intraspecific hybridization (Fitzpatrick, 2012).

This study presents the assembled genomes of both a female and a male white perch (NCBI Taxonomy ID: 46260), and a high-quality genome annotation using RNA-seq data for the female assembly. We assembled genomes for both sexes to support future studies of how sex might influence contaminant sensitivity and hybridization dynamics. To date, only a mitogenome for the white perch exists (Bian et al., 2016), with no assembled nuclear genome. The closest assembled genomes are those of the white bass (*Morone* chrysops, NCBI Taxonomy ID: 46259; NCBI Accession: GCA_019097615) and the striped sea-bass (*Morone saxatilis*, NCBI Taxonomy ID: 34816; NCBI Accession: GCA_004916995), which diverged from the white perch 13.2 and 14.4 million years ago, respectively (Sanciangco et al., 2016). These genomes will provide a crucial resource for studying the ecological and evolutionary responses to environmental contaminants, understanding the species’ invasion biology, and facilitating studies on quantifying its hybridization with congeneric species.

## Methods

### Biological materials

Adult white perch (NCBI Taxonomy ID 46260) were collected in June 2022 from the Mullica River (Great Bay, New Jersey, USA). The Mullica River has been used as a reference area to support remedial investigations of industrial waterways (e.g. Ludwig & Iannuzzi, 2005; Iannuzzi et al., 2008) and the river’s ecology has been well-studied (Able et al., 1996, 1999, 2020; Kennish et al., 2004; Thompson et al., 2022). Fish were collected from areas of low salinity, consistent with their classification within this system as freshwater spawning residents (Nickerson et al., 2018). Fish were collected with approval from New Jersey Fish & Wildlife, Marine Fisheries Administration (Permit number: 2022-1833). Live fish were transported to the NOAA James J. Howard Marine Sciences Laboratory (Sandy Hook, New Jersey, USA) and were maintained in holding tanks at approximately 2 to 5 ppt salinity and 12 °C for < 48 h prior to euthanasia.

For the genome assembly, one adult male perch >150 mm and one adult female perch >150 mm were gently netted from the tank, and immediately euthanized using an overdose of 300 mg/ml of MS-222 in water. Blood was obtained from the caudal vein (500 µl) using a capillary tube and was immediately frozen in liquid nitrogen. Care was taken to avoid contamination of the blood with interstitial fluid. We also dissected approximately 1 g of muscle fillet from dorsal/epaxial muscles, which was frozen in liquid nitrogen. Tissues were stored at −80 °C prior to DNA extraction.

For the genome annotation, we extracted ovary tissue from female white perch (*n* = 12) and from 28 days post fertilization (dpf) larvae (*n* = 120). Larvae were obtained from eggs spawned (in the laboratory) by adults collected from the Mullica River. Ovary tissue was dissected and frozen in liquid nitrogen. Five larvae were pooled into a single tube prior to homogenization and frozen in liquid nitrogen, resulting in a total of *n* = 24 larval samples. All samples were stored at −80 °C prior to RNA extraction. All tissues were shipped on dry ice to the University of Oregon (Eugene, Oregon, USA) for DNA and RNA extraction, and were sequenced at the University of Oregon and Floragenex Inc. (Eugene, Oregon, USA).

### Nucleic acid library preparation

DNA was extracted from the blood and muscle tissue using the PacBio NanoBind Tissue Kit (PacBio, CA, USA). DNA was checked for quality using a Qubit™ dsDNA HS (High Sensitivity) Assay kit and via gel electrophoresis. Long-read libraries were generated using the PacBio SMRTbell Prep Kit 3.0. HMW DNA was sheared to 15 to 18 Kb, and SMRTbell adapters were attached according to the PacBio protocol. Prior to sequencing, the libraries underwent 0.45x Ampure bead size selection to remove fragments <3 kb. Library concentration was measured with a Qubit fluorometer (Thermo Fisher Scientific, Waltham, MA, USA), and fragment size distribution was assessed with the Agilent Fragment Analyzer (Agilent Technologies, Santa Clara, CA, USA). A total of 218 Gb of unique molecular data (1106 Gb of raw data) using two SMRT Cells 8M (per sex) was generated on a Pacbio Sequel II. PacBio HiFi data were filtered to retain only those reads with *Q* > 20.

RNA was extracted using the Zymo Direct-zol RNA kit (Irvine, California, USA), following the manufacturer’s instructions. RNA quality and quantification were checked using a Qubit™ RNA HS Assay kit and using the Agilent Fragment Analyzer. Libraries were prepared using the KAPA mRNA HyperPrep Kit (KK8581). Input RNA of 10 to 1000 ng was normalized based on the extraction quantification. Samples were PolyA selected and fragmented, followed by cDNA synthesis and the ligation of adapters. Libraries were amplified and then quantified using the Agilent Fragment Analyzer. qPCR was also run to determine final library sequencing concentrations. Libraries were sequenced on a NovaSeq 6000 (SP300 chip) with 150 bp paired-end sequencing.

### Genome assembly

Table 1 lists the programs and non-default parameters used in the assembly of the genomes. HiFi reads were assembled using Hifiasm v0.19 (Cheng et al., 2021). Assembly statistics were computed using quast-lg v5.0.2 (Mikheenko et al., 2018). Assembly completeness was assessed using BUSCO v5.2.2 (Simão et al., 2015), using the actinopterygii catalog (*n* = 3,640) of orthologous protein-coding genes from OrthoDb v10 (Kriventseva et al., 2019). To further assess contiguity, we calculated the percentage of the total assembly contained in the first *n* = 24 contigs, as the reported karyotype of the white perch is 2*n* = 48 (Kerby, 1972). Assessment of base level accuracy (QV) and *k*-mer completeness was performed by counting 21-mers with meryl in Merqury v1.3 (Rhie et al., 2020), using the PacBio HiFi reads used to generate the assemblies. The mitogenome was assembled using MitoHifi v3.2 (Uliano-Silva et al., 2023) using the contigs of the female assembly, and the previously published mitochondrial sequence of the white perch (KU641485; Bian et al., 2016) as the reference mitogenome.

**Table 1.** List of programs used for the assembly and quality assessment of the two *Morone americana* genomes.

Purpose	Program and parameters version	Version
**Assembly**		
*k*-mer counting	Jellyfish (*k* = 15, *k *= 21, *k* = 31)	v2.3.0
*k*-mer counting	Meryl (*k *= 21)	v1.3
*k*-mer estimation of genome size and heterozygosity	GenomeScope (*k* = 15, *k *= 21, *k* = 31, p = 2, l = 41)	v2.0
de novo nuclear genome assembly	Hifiasm	v0.19
Mitogenome assembly	MitoHifi (--circular-size 17000 -o 2 -p 80)	v3.2
**Genome quality assessment**		
Assembly statistics	quast-lg	v5.0.2
Assembly completeness	BUSCO (-m genome, -l actinopterygii_odb10)	v5.0.2
Assembly completeness	Merqury	v1.3

We generated *k*-mer databases from the PacBio HiFi reads using Jellyfish v2.3.0 (Marçais & Kingsford, 2011), exploring *k*-mer sizes of *k* = 15, *k* = 21 and *k* = 31. *K*-mer databases were used to assess the haploid genome size and heterozygosity with GenomeScope v2 (Ranallo-Benavidez et al., 2020). Due to an observed low heterozygosity, the default model was not able to differentiate between the homozygous and heterozygous peaks. We therefore adjusted the *lambda* (-l) parameter, which sets the initial guess for the average *k*-mer coverage of the sequencing data, to assess the estimated genome span more accurately. To further assess the observed low heterozygosity, we also performed *k*-mer profiling on short-read Illumina data from the white bass (BioProject PRJNA478192), and from wild populations of the more distantly related European sea bass (*Dicentrarchus labrax*: BioProject: PRJEB40423).

### Gene prediction and functional annotation

Assemblies were assessed for repeat content using RepeatModeler v2.0.2 (Smit & Hubley, 2015) and RepeatMasker v4.1.0 (Smit et al., 2015) (http://www.repeatmasker.org). RepeatMasker was run with rmblastn v2.10.0 using Dfam v3.1 (Storer et al., 2021) as the repeat database. The RepeatMasker summary table was created using buildSummary.pl to incorporate species-specific repeats.

Functional annotation was performed using BRAKER3 (Hoff et al., 2019; Brůna et al., 2024; Gabriel et al., 2024), using the ovary and larvae RNA-seq data, and protein data derived from the Vertebrata partition of OrthoDB version 11 (Kuznetsov et al., 2023) as evidence. RNA-seq data were mapped using HISAT2 (Kim et al., 2019). BRAKER3 uses the GeneMark-ETP pipeline (Brůna, Lomsadze, et al., 2024) to assemble the RNA-seq data using StringTie2 (Kovaka et al., 2019). AUGUSTUS (Stanke et al., 2006, 2008) was trained on the high-quality genes predicted from GeneMark-ETP, using DIAMOND (Buchfink et al., 2015) to filter out redundant gene structures. The predicted gene sets were combined using TSEBRA (Gabriel et al., 2021). BRAKER3 was called using the --busco_lineage actinopterygii_odb10 option, which uses BUSCO (Simão et al., 2015), miniprot (Li, 2023) and compleasm (Huang & Li, 2023). The annotation files were converted using agat_sp_keep_longest_isoform.pl and agat_sp_extract_sequences.pl with AGAT (Dainat, 2024). Quality and completeness of the annotation was assessed using BUSCO (Simão et al., 2015) in protein mode against the actinopterygii_odb10 dataset (*n* = 3,640 orthogroups), OMArk against the ancestral clade Eupercaria (*n* = 15,616 hierarchical orthologous groups) and by computing statistics using agat_sp_statistics.pl in AGAT (Dainat, 2024). Gene annotations were visually assessed for support using IGV v2.16.1 (Thorvaldsdóttir et al., 2013).

Functional annotation of the final set of protein-coding genes was performed using InterProScan v5.72-103 (Jones et al., 2014; Blum et al., 2021) and EggNOG-mapper v2 (Cantalapiedra et al., 2021) against the eggNOG v5 database (Huerta-Cepas et al., 2019) using an e-value of 0.001, including predicted protein domains, gene names, gene descriptions, Gene Ontology (GO) and Kyoto Encyclopedia of Genes and Genomes (KEGG) Terms. Results were incorporated using agat_sp_manage_attributes.pl and agat_sp_manage_functional_annotation.pl in AGAT (Dainat, 2024).

## Results

We assembled a female white perch genome from 4,631,274 quality-filtered HiFi reads, resulting in an assembly spanning 694 Mb, comprising 264 contigs (average 84X coverage; N50: 24.8 Mb; Table 2), of which 100 contigs are > 10 Mb. The largest 24 contigs in the female assembly account for 85% of the total genome span, which range from 13 Mb to 32 Mb. Assessment of completeness and accuracy using Merqury revealed a *k*-mer completeness of 98.80 and a base-pair QV accuracy of 59.10. BUSCO assessment revealed a high completeness of 98.7%.

**Table 2.** Assembly statistics for the female and male assemblies of the white perch (*Morone americana*) and comparison to the two closest species with genome assemblies: the striped sea-bass (*Morone saxatillis*) and the white bass (*Morone chrysops*).

Assembly Statistics	White perch female	White perch male	Striped sea-bass	White bass
	(*M. americana*)	(*M. americana*)	(*M. saxatillis*)	(*M. chrysops*)
Genebank accession	GCA_965119275	GCA_965119265	GCA_004916995.1	GCA_019097615.1
Genome size (Mb)	693.3	688.6	598.1	640.4
Number of contigs / scaffolds	263	264	629	12,898
Longest contig (Mb)	32.09	32.23	31.20	32.21
N50 (Mb)	24.88	26.44	25.90	28.00
L50	13	12	11	11
Merqury completeness ^a^	98.7908	98.8775	NA	NA
Merqury QV ^a^	59.0529	58.8351	NA	NA
BUSCO ^b^	C:98.7% [S:97.8%, D:0.9%], F:0.3%, M:1.0%	C:98.8% [S:98.1%, D:0.7%], F:0.3%, M:0.9%	C:95.5% [S:94.8%, D:0.7%], F:1.8%, M:2.7%	C:98.1% [S:97.6%, D:0.5%], F:0.8%, M:1.1%

^a^Merqury statistics are based on *k* = 21.

^b^BUSCO statistics were calculated using actinopterygii_odb10 (*n* = 3640 orthogroups). Complete BUSCOs (C). Complete and single-copy BUSCOs (S). Complete and duplicate BUSCOs (D). Fragmented BUSCOs (F). Missing BUSCOs (M).

We assembled a male white perch genome from 4,476,817 quality-filtered reads, resulting in an assembly span of 688 Mb, comprising 265 contigs (average 80X coverage; N50: 26.4 Mb; Table 2), of which 116 contigs are > 10 Mb. The largest 24 contigs in the male assembly account for 89% of the total genome span, ranging from 14 Mb to 32 Mb. Assessment of completeness and accuracy using Merqury revealed a *k*-mer completeness of 98.88 and a base-pair QV of 58.84. BUSCO assessment revealed a high completeness of 98.7%.

We also obtained a complete (but not circularized) mitogenome for the species (17,848 bp), which contained all 37 mitochondrial vertebrate genes. Like the previously published mitogenome, and several other Moronidae species, we found that the ND6 gene is located within the control region (Williams et al. 2012; Tian et al. 2014; Bian et al. 2016). The mitogenome can be accessed on GenBank under the accession number PV254232.

**Fig. 2.**
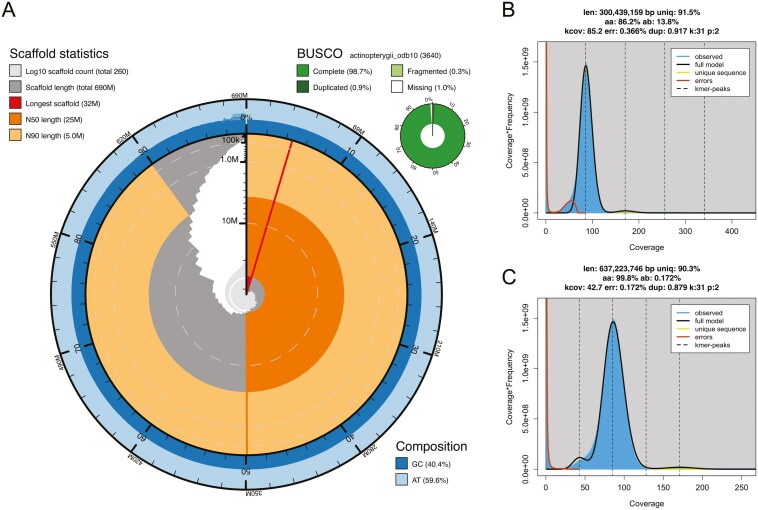
Genome assembly metrics for the female white perch, *Morone americana*. A) Snail plot summarizing the assembly statistics (also presented in Table 1). The plot circle represents the full size of the assembly. The distribution of contig lengths is shown in dark gray with the plot radius scaled to the longest contig in the assembly (32 Mbp, in red). The orange and pale-orange arcs represent the N50 (24.88 Mbp) and the N90 (5 Mbp) statistics, respectively. The pale gray spiral shows the cumulative contig count on a log scale with white scale lines showing successive orders of magnitude. The blue and pale-blue areas around the outside of the plot show the distribution of GC, AT, and N percentages in the same bins (*n* = 1,000) as the inner plot. A summary of complete, fragmented, duplicated, and missing BUSCO genes in the actinopterygii_odb10 database is shown in the top-right. (B) and (C) show the *k*-mer profiles generated in GenomeScope, using *k* = 21. B) The default model converges incorrectly when it is fitted to very homozygous *k*-mer spectra, as demonstrated here when only one peak is visible. This causes an underestimation of haploid genome size (300 Mb) and an overestimation of heterozygosity (13.8%). C) After correcting the coverage prior (the lambda parameter), the *k*-mer profile shows a more realistic haploid genome size (637 Mb) and heterozygosity (0.17%). For the male assembly statistics and *k*-mer profiles, see Supplementary Figure S1.

*K*-mer assessment revealed low heterozygosity in both genomes (Fig. 2 for the female *k*-mer profiles; see Supplementary Figure S1 for the male *k*-mer profiles). After correcting the GenomeScope model for low heterozygosity, genome size *k*-mer estimates ranged from 621 Mb (*k *= 15) to 637 Mb (*k* = 31) for the female, and 619 Mb (*k *= 15) to 635 Mb (*k* = 31) for the male assembly (see Supplementary Table S1). To examine whether low heterozygosity is a biological or technical artifact, we also performed *k*-mer analysis on the white bass (see Supplementary Figure S2) and the European seabass (see Supplementary Figure S3). We identified a lack of bimodality in the *k*-mer profiles in both species indicating that the observed low heterozygosity is likely a biological feature of Moronidae.

Applying de novo repeat identification, we identified 24.5% of the female assembly and 23.6% of the male assembly, respectively, as repetitive. This proportion is similar to the repeat content reported for the striped sea-bass (22%) and the European sea-bass, *Dicentrarchus labrax* (21.47%; Tine et al. 2014). Of the repeat families that could be identified, the most common repeat family was DNA transposons (female: 6.37%; male: 6.83%) as has been commonly observed in teleosts (Reinar et al., 2023), followed by LINEs (female: 3.31%; male: 3.21%). The male genome contained fewer SINEs (0.76%) in comparison to the female genome (1.13%), but *Alu* repeats were only present in the male genome, albeit at a low frequency. See Supplementary Table S2 for the full details of the identified repeat families.

Gene annotation of the female assembly predicted a total of 20,699 protein-coding genes, with a high BUSCO completeness score: 98.9% and a good OMArk completeness score: 87.12%. In OMArk, the proteome showed a 91.02% consistent lineage assessment, and no contamination was identified. Functional annotation identified protein domain homology for 20,406 genes, with 16,187 genes having gene names and 11,977 having gene descriptions. Full statistics on the structural and functional annotation can be found in Supplementary Table S3.

## Discussion

We generated highly contiguous genome assemblies for a female and a male white perch. The resulting assemblies are more contiguous (fewer contigs) and are more complete (BUSCO scores) compared with the other two published *Morone* genomes assemblies of the striped sea-bass and white bass. Even without the use of chromosome conformation capture technologies, or alternative scaffolding strategies, we found that a large proportion of both assemblies were contained within the first 24 contigs, highlighting the power of high-accuracy long-read sequencing technologies (Wenger et al., 2019) and substantial improvements in assembly algorithms (Espinosa et al., 2024).

We also generated a high-quality genome annotation for the female white perch. This annotation showed a very high BUSCO completeness (99%) but a comparatively lower OMark completeness score (87%). This discrepancy likely arises from differences in the lineage-specific datasets used by each of the programs. BUSCO results were analyzed using the compact actinopterygii_odb10 (*n* = 3,640), which comprises only highly conserved single-copy orthologs tailored specifically for ray-finned fishes. OMark uses a broader dataset and evaluates completeness against a larger set (*n* = 15,616) of hierarchical orthogroups (HOGs) and covers a wider phylogenetic range, including less conserved or lineage-specific genes (Nevers et al., 2025). Many of these orthogroups may be absent in our genome annotation due to species-specific gene losses, incomplete genome assembly, or annotation gaps caused by the tissue-limited transcriptomic data used.

The limited bimodality of the *k*-mer spectra of both the male and female genome indicates low heterozygosity in the white perch. Comparison of the *k*-mer profiles with those generated using short-read Illumina data derived from white bass and the European sea bass suggests that the observed low heterozygosity appears to be a biological feature of Moronidae, but more genomic data is needed to further understand the reason for low genome-wide heterozygosity in this family. Correcting the default parameters of the GenomeScope model substantially improved estimates of the genome properties for the white perch and should be considered for other species showing evidence of low heterozygosity in their *k*-mer profiles.

Overall, the generated genome assemblies for both sexes of the white perch will provide a valuable resource for further developing the species as a bioindicator, quantifying the evolutionary processes underpinning the species’ invasiveness, and creating genomics resources for quantifying intraspecific hybridization. The generated assemblies for both sexes will aid future explorations into sex-specific responses to contaminants, sex-linked genes, and potentially sex-biased rates of hybridization. Given the widespread distribution of the white perch, these genomes will also permit population and conservation genomic studies of the species across its native and non-native range.

## Supplementary Material

esaf034_suppl_Supplementary_Figures

esaf034_suppl_Supplementary_Tables

## Data Availability

Genome assemblies and accompanying raw PacBio data for this project are available on the European Nucleotide Archive (ENA) under the study accession PRJEB63122. The female white perch (Morone americana) assembly can be found under the genome accession number GCA_965119275. The male white perch (Morone americana) assembly can be found under the genome accession number GCA_965119265. The mitochondrial sequence is available on NCBI GenBank under the accession number PV254232. Structural and functional gene annotation files are available on GitHub (https://github.com/josieparis/Morone_americana-assembly-annotation), which has been archived under the Zenodo DOI: 10.5821/zenodo.14772921
