# Supplementary material for: Two genomes of the white perch (Morone americana), an ecologically important teleost
Source: J Hered. 2025 May 30;116(6):846–53. doi: 10.1093/jhered/esaf034 (PMC12584590; doi:10.1093/jhered/esaf034)
Supplement: esaf034_suppl_Supplementary_Figures [file esaf034_suppl_supplementary_figures.pdf]

Supplementary Figures for:

Two genomes of the white perch (*Morone americana*), an ecologically important teleost

Authors

Josephine R. Paris, Megan A. Criss, Jessica L. Walsh, Joan Ferrer Obiol, Christopher S. Murray, Jason Q. Boone, Ann M. Petersen

Corresponding Author: Josephine Paris (parisjosephine@gmail.com)

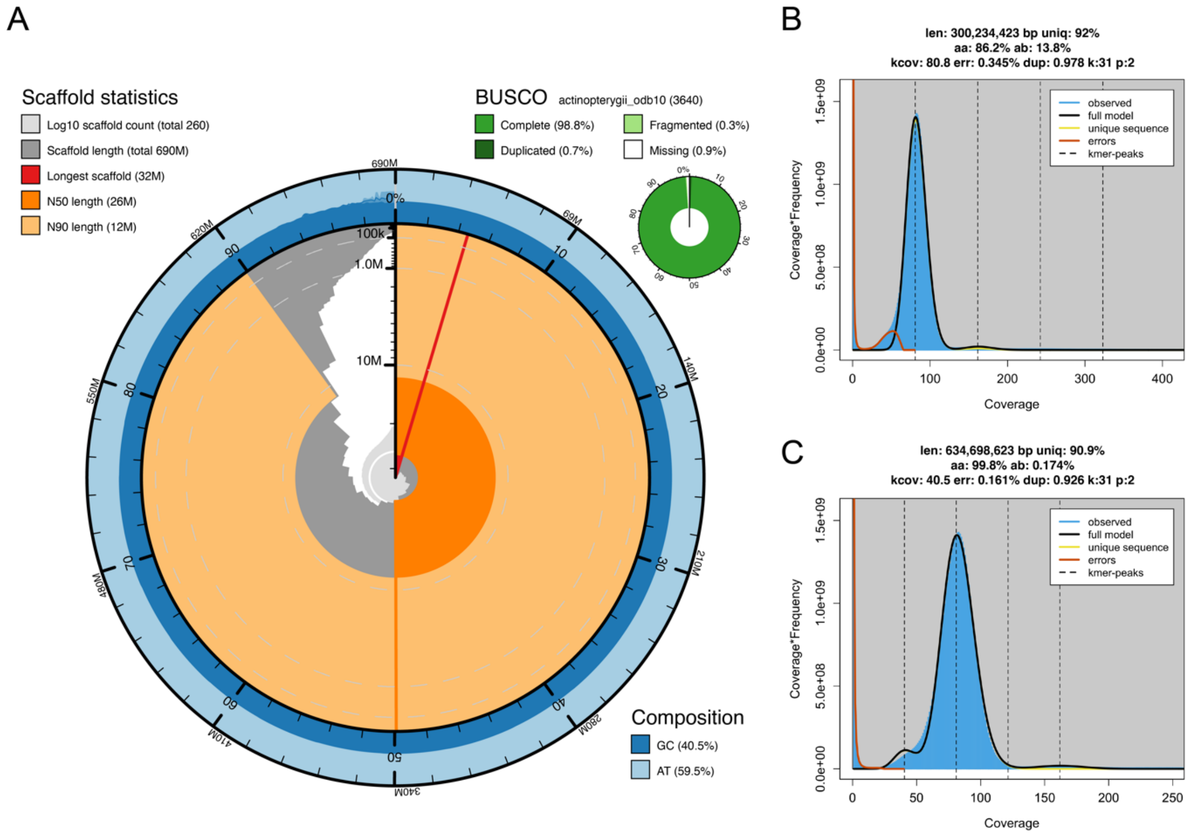

Supplementary Figure 1. **Genome assembly metrics for the male white perch (*Morone americana*)**. (**A**) Snail plot summarizing the assembly statistics (also presented in Table 1). The plot circle represents the full size of the assembly. The distribution of contig lengths is shown in dark grey with the plot radius scaled to the longest contig in the assembly (32 Mbp, in red). The orange and pale-orange arcs represent the N50 (26 Mbp) and the N90 (12 Mbp) statistics, respectively. The pale grey spiral shows the cumulative contig count on a log scale with white scale lines showing successive orders of magnitude. The blue and pale-blue areas around the outside of the plot show the distribution of GC, AT and N percentages in the same bins (n = 1,000) as the inner plot. A summary of complete, fragmented, duplicated and missing BUSCO genes in the actinopterygii_odb10 database is shown in the top-right. (**B**) and (**C**) show the *k*-mer profiles generated in GenomeScope, using *k* = 21. (**B**) shows that the default model converges incorrectly when it is fitted to very homozygous *k*-mer spectra, as demonstrated here when only one peak is visible. This causes an underestimation of haploid genome size (300 Mb) and an overestimation of heterozygosity (13.8%). (**C**) After correcting the coverage prior (the *lambda* parameter), the *k*-mer profile shows a more realistic haploid genome size (635 Mb) and heterozygosity (0.17%).

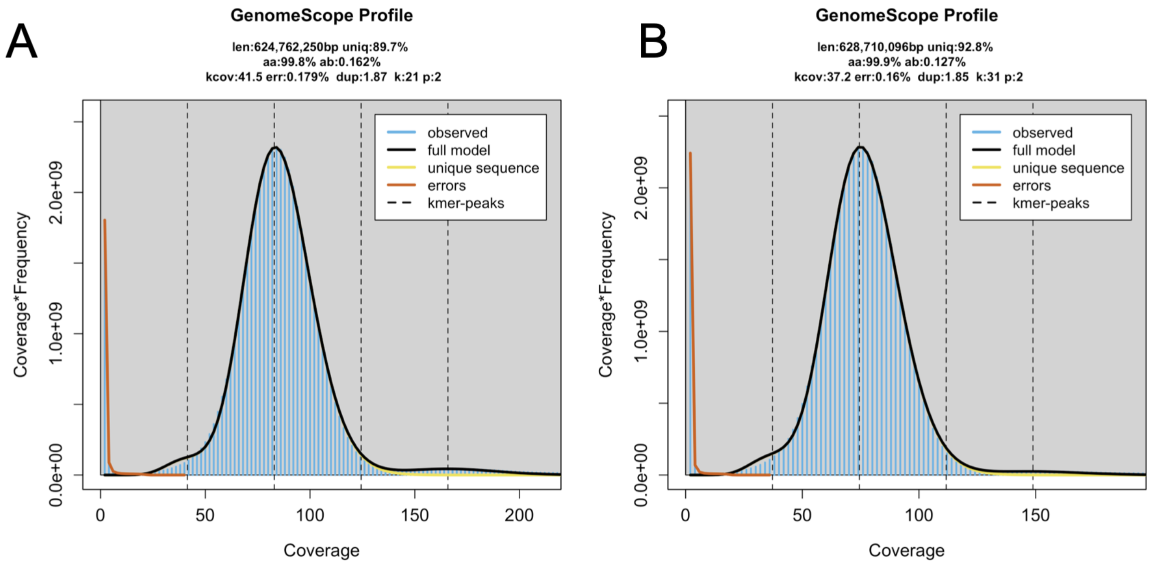

Supplementary Figure 2. *K*-mer profiles generated in GenomeScope2 for the white bass (*Morone chrysops*). (**A**) Default model for k = 21 showing a haploid genome size of 625 Mb and a heterozygosity of 0.16%. (**B**) Default model for k = 31 showing a haploid genome size of 629 Mb and a heterozygosity of 0.13%. *K*-mer profiles were generated using Illumina short-read data accessed on the ENA (BioProject: PRJNA478192).

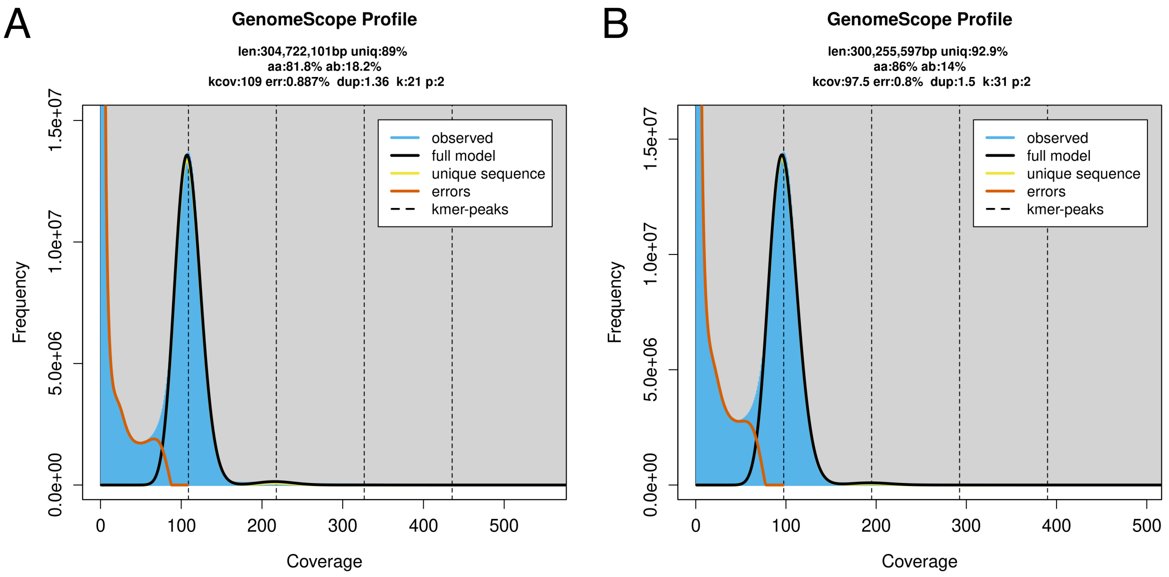

Supplementary Figure 3. *K*-mer profiles generated in GenomeScope2 for the European seabass (*Dicentrarchus labrax*). (**A**) Default model for *k* = 21 showing a haploid genome size of 304 Mb and a heterozygosity of 18.2%. (**B**) Default model for *k* = 31 showing a haploid genome size of 300 Mb and a heterozygosity of 14%. *K*-mer profiles were generated using Illumina short-read data accessed on the ENA (BioProject: PRJEB40423).
